# Identification of potential biomarkers for aging diagnosis of mesenchymal stem cells derived from the aged donors

**DOI:** 10.1186/s13287-024-03689-1

**Published:** 2024-03-22

**Authors:** Miao Hao, Hongyu Jiang, Yuan Zhao, Chunyi Li, Jinlan Jiang

**Affiliations:** 1 Scientific Research Center, China-Japan Union Hospital of Jilin University, 130000 Changchun, Jilin China; 2 Life Spring AKY Pharmaceuticals, 130000 Changchun, Jilin China; 3 Institute of Antler Science and Product Technology, Changchun Sci-Tech University, 130000 Changchun, Jilin, China

**Keywords:** Bioinformatics, Diagnosis, Mesenchymal stem cells, Senescence, Tumorigenesis

## Abstract

**Background:**

The clinical application of human bone-marrow derived mesenchymal stem cells (MSCs) for the treatment of refractory diseases has achieved remarkable results. However, there is a need for a systematic evaluation of the quality and safety of MSCs sourced from donors. In this study, we sought to assess one potential factor that might impact quality, namely the age of the donor.

**Methods:**

We downloaded two data sets from each of two Gene Expression Omnibus (GEO), GSE39035 and GSE97311 databases, namely samples form young (< 65 years of age) and old (> 65) donor groups. Through, bioinformatics analysis and experimental validation to these retrieved data, we found that MSCs derived from aged donors can lead to differential expression of gene profiles compared with those from young donors, and potentially affect the function of MSCs, and may even induce malignant tumors.

**Results:**

We identified a total of 337 differentially expressed genes (DEGs), including two upregulated and eight downregulated genes from the databases of both GSE39035 and GSE97311. We further identified 13 hub genes. Six of them, *TBX15, IGF1, GATA2, PITX2, SNAI1* and *VCAN*, were highly expressed in many human malignancies in Human Protein Atlas database. In the MSCs in vitro senescent cell model, qPCR analysis validated that all six hub genes were highly expressed in senescent MSCs. Our findings confirm that aged donors of MSCs have a significant effect on gene expression profiles. The MSCs from old donors have the potential to cause a variety of malignancies. These *TBX15, IGF1, GATA2, PITX2, SNAI1, VCAN* genes could be used as potential biomarkers to diagnosis aging state of donor MSCs, and evaluate whether MSCs derived from an aged donor could be used for therapy in the clinic. Our findings provide a diagnostic basis for the clinical use of MSCs to treat a variety of diseases.

**Conclusions:**

Therefore, our findings not only provide guidance for the safe and standardized use of MSCs in the clinic for the treatment of various diseases, but also provide insights into the use of cell regeneration approaches to reverse aging and support rejuvenation.

**Supplementary Information:**

The online version contains supplementary material available at 10.1186/s13287-024-03689-1.

## Background

Mesenchymal stem cells (MSCs) are a class of stromal cells, including multipotent stem cells, progenitors, and differentiated cells. MSCs are isolated primarily from bone marrow, adipose tissue, and umbilical cord, which have the ability to differentiate into bone, cartilage, or fat cells [1]. Current criteria for MSCs isolation yield heterogeneous, non-clonal cultures of stromal cells, including stem cells with diverse multipotential properties, committed progenitors, and differentiated cells [2, 3]. As promising new therapeutic strategy, MSCs-based therapy is under investigation for treatment of many conditions, including inflammatory, ischemic and neurodegenerative diseases [4]. Although human bone-marrow derived MSCs have both proliferative capacity and chondrogenic potential, the possibility dysfunction, accelerated aging, and malignancy are considered as potential threats to clinical applications. It is well known that the regenerative capacity of MSCs depends upon the cell source, donor age, and culture conditions [5]. For example, many studies have suggested that expansion could change the lipid composition of MSCs, such as glycosphingolipids and signaling lipids [6–9]. For example, there was evidence of differences in the glycerophospholipids profiles and in the expression of genes related to lipid metabolism and immunomodulation of MSCs from young and old donors during sequential expansion [5].

As they age, the ability of MSCs to self-renew and to differentiate gradually declines [10]. In this respect, research has suggested that MSCs play an important role in skeletal homeostasis by serving as a reservoir of osteoblast precursors and studies have linked age-related bone loss to defects in the function of MSCs including loss of proliferative and differentiation potential and an increased rate of senescence [11]. Therefore, the age of the MSC donor might have an important influence on its cell function and value in cell therapy, although there is still controversy about the effect of donor age on human adipose tissue MSCs [12]. Thus, the effect of donor age on MSCs in terms of the differential expression and function of their genes is still inconclusive. The replicative capacity of MSCs decrease with donor age, while an increase apoptotic cells with positive staining for senescence-associated β-galactosidase-positive staining [13]. For example, Age negatively affects the capacity of MSC harvesting and proliferation, and even the capacity for osteogenic differentiation [14]. It has been reported that MSCs from older donors not only have a susceptibility to produce more nitrous oxide and reactive oxide species, but also have a reduced ability to catalyze superoxide radicals [15, 16]. In addition, bone marrow-derived MSCs from young donors migrate more efficiently to lesions and differentiate into neuronal cells compared to older bone marrow-derived MSCs for treating rats with central nervous system demyelination [17]. Currently, transcriptomic and microarray analyses have been used widely to help identify new biomarkers to improve diagnosis and treatment of a variety of diseases [18], so that through a combination of microarray and bioinformatics analyses, it is possible to explore potential key genes and pathway networks that may be related to the development of diseases.

This work provided insights into mechanisms of development of aging MSCs at the transcriptome level and explores potential biomarkers of the early diagnosis of aging MSCs in the clinical settings. Our study helps to standardize the collection criteria of donors of MSCs, and provides guidance and reference for the application of MSCs to treat various diseases in the clinic.

## Methods

### Microarray data collection

The microarray datasets of human MSCs from young and old donors were downloaded from the Gene Expression Omnibus (GEO) database (http://www.ncbi.nlm.nih.gov/geo) [19]. The samples from two datasets were divided into those from young (< 65 years of age) and old (> 65) donor groups. The GSE39035 dataset contained the gene expression profiles four old donors and four young donors, with eight samples from each. In the GSE97311 dataset, there were three young samples and four old samples of MSCs available for analysis (Table 1). Because these gene expression profiles originated from a free open-access database on the internet, our research did not require Ethics Committee approval [20].

**Table 1:** Information for selected microarray datasets

GEO accession	Platform	Samples (Total number)	Samples		PMID
			young	old	
GSE39035	GPL13607	16	8	8	23271708
GSE97311	GPL10558	18	3	4	30885246

Annotation: GPL13607: Agilent-028004 SurePrint G3 Human GE 8 × 60 K Microarray (Feature Number version); GPL10558: Illumina HumanHT-12 V4.0 expression beadchip

### PCA of gene expression

In the present study, the quality of each microarray data was evaluated by conducting PCA [21] prior to analysis of the DEGs. R language was used to evaluate the PCA of gene expression, resulting in identification of the DEGs that could be considered as variables that showed the differences between young and old samples of MSCs in GSE39035 dataset and GSE97311 datasets.

### Data normalization and identification of DEGs

The original files that were downloaded from the GEO database were pre-processed and normalized using the R (3.6.3) package, which is an efficient analysis method in bioinformatics [22–24]. The selected criteria were set as adjusted-*p* < 0.05 and |log_2_FC| >1. After we utilized these screening conditions, two sets of DEGs were identified, then we put these DEGs into an online analysis tool Venn (http://bioinformatics.psb.ugent.be/webtools/Venn/) to obtain up- and down-regulated intersection genes, respectively. To better visualize these DEGs, R software was used to make heatmaps and volcano plots. These intersecting genes were used for subsequent analysis.

### Identification of tissue/organ‑specific expressed genes

To understand the tissue/organ-specific expression of these DEGs, the online tool BioGPS (http://biogps.org/) was used to analyse the tissue distribution [25]. The screening criteria were as follows: (1) transcripts that mapped to a single organ system with an expression value of > 10 multiples of the median, and (2) second-most-abundant tissue expression was no more than a third as high [26]. The genes obtained by these criteria were considered to be tissue-specific genes.

### Functional enrichment analyses for DEGs

The Gene Ontology (GO) [27] classification, which contains molecular functions (MF), biological processes (BP), and cellular component (CC) were performed on the intersecting DEGs using the R package. P-values of < 0.05 were defined as statistically significant. Reactome pathway enrichment analyses were performed using Reactome Pathway Database (https://reactome.org/). The intersecting DEGs were uploaded to the Reactome Database.

### Construction of protein–protein interaction (PPI) network and identification of hub genes

To further explore the interaction among the genes obtained above, we used the Search Tool for the Retrieval of Interacting Genes (STRING) 11.0 (http://string-db.org/) [28] to construct a PPI network. The PPI network was constructed based on all DEGs by the online tool STRING (https://string-db.org/) with a filter condition (combined score > 0.4) [29]. In the network outcome, the nodes represented the proteins, while the lines represented the interactions between proteins [30, 31]. Cytoscape software (version 3.9.1) was performed to construct the network plots after PPI relationship. The Cytoscape plugin was used for topological analysis of the nodes in the network. Then, all the results were intersected to obtain the final hub genes.

### Immunohistochemistry (IHC) staining

To evaluate hub genes expression at the protein level, IHC images in normal and five tumors tissues, including ovarian cancer, lung cancer, prostate cancer, breast cancer and liver cancer, were downloaded from the HPA (http://www.proteinatlas.org/) and analyzed.

### Cell culture

Human bone-marrow derived mesenchymal stem cell lines (36-year-old female) were purchased from OriCell®. MSCs were cultured in basal medium supplemented with 10% fetal bovine serum in a humidified atmosphere of 5% CO_2_ at 37^o^C.

### Cell viability assay

The cells were seeded in 96-well plates. Then, the cells were treated with different concentrations of H_2_O_2_ for 2 h. After 48 h of continued incubation, 10 µL of CCK-8 reagent (Dojindo) was added into each well. After 2 h, the OD 450 nm value was determined by a microplate reader. All experiments were performed in triplicate at least.

### Quantitative real-time PCR (qPCR)

To assessed MSC senescence model in vitro and verify the expressions of hub genes, qPCR analysis was performed. Total RNA was isolated using the TRIzol reagent (Invitrogen, USA). Reverse transcription was performed using PrimeScript™ RT reagent Kit (RR047A, TaKaRa, Japan) according to the manufacturer’s instructions. DNA was isolated using Wizard® HMW DNA Extraction Kit (Promega, USA) according to the manufacturer’s instructions. Relative mRNA or DNA expressions were quantified by PCR with SYBR Green PCR Kit (Roche, Germany) and normalized to 36B or GAPDH. The primers detail was listed in Supplementary Table 1. The cycling parameters were 40 cycles of 95^o^C for 15s, 60^o^C for 15s, and 72^o^C for 30s. Relative gene level was normalized to 36B or GAPDH and calculated according to the Livak method (2^−ΔΔCt^) [32]. The experiments were independently repeated three times.

### Statistics analysis

All the data were analyzed by SPSS 22.0 (SPSS, Inc, Chicago, IL, USA) software. The results were expressed as mean ± standard deviation (SD). Using SPSS 22.0, we constructed receiver operating characteristic (ROC) curves and calculated the area under the curve (AUC) of the hub genes to compare the AUC as the index of the model. These results showed the diagnostic efficiency of genes. P-values < 0.05 were regarded as statistically significant.

## Results

### Validation of the datasets and identification of DEGs

**Fig. 1:**
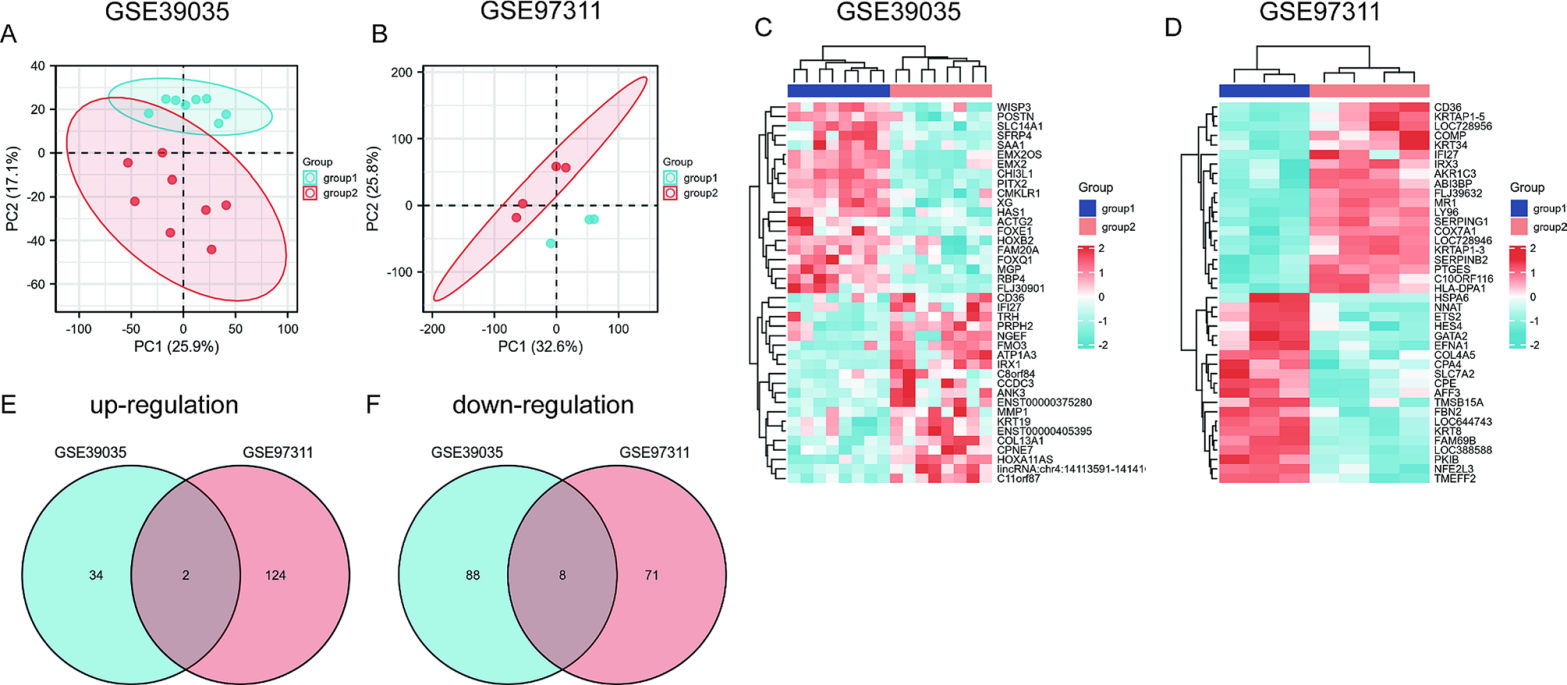
Principal component analysis (PCA) and Screening of DEGs. A. PCA of DEGs between samples of MSCs from young (< 65 years of age) and old (> 65) donors in the GSE39035 dataset. B. PCA of DEGs between samples of MSCs from young (< 65 years of age) and old (> 65) donors in the GSE97311 dataset. C. Heatmap of DEGs in samples of MSCs from young (< 65 years of age) and old (> 65) donors in the GSE39035 dataset. D. Heatmap of DEGs in samples of MSCs from young (< 65 years of age) and old (> 65) donors in the GSE97311 dataset. Red rectangles represent a high level of expression, and blue rectangles represent a low level of expression. Group1 from young (< 65 years of age) and Group2 from old (> 65) donors. E. Venn diagram of intersecting co-expressed upregulated genes (DEGs) from the GSE39035 and GSE97311 datasets. logFC > 1, and adj.*P* < 0.05. F. Venn diagram of intersecting co-expressed downregulated genes (DEGs) from GSE39035 and GSE97311 datasets. logFC <-1, and adj.*P* < 0.05

To investigate intra-group data repeatability, PCA was employed to reveal the data distribution in each sample to ensure that the data structure (young and old) was appropriate (accurate and reliable). As shown in Fig. 1A, the evaluation showed that the data from GSE39035 could be unambiguously divided into two groups based on age. Likewise in the GSE97311 dataset (Fig. 1B), the data could be divided into young and old groups according to age.

There were 132 DEGs identified in the GSE39035 dataset, including 36 upregulated (UP) genes and 96 downregulated (DOWN) genes. Heatmap and volcano plot analyses were used to visualize these DEGs (Fig. 1C and S1A). In the GSE97311 dataset, 205 DEGs were identified, including 126 UP genes and 79 DOWN genes (Fig. 1D and S1B).

Using a Venn Diagram online, 337 intersecting genes of the two datasets were obtained, including two co-expressed UP and eight co-expressed DOWN genes (Fig. 1E and F). The details of these co-expressed genes are also shown in Table 2.

**Table 2:** Common upregulated and downregulated DEGs in GSE39035 and GSE97311

Gene	GSE39035		GSE97311		regulation
	Log FC	Adj.P	Log FC	Adj.P	
PORCN	1.0217	0.0300	1.8303	0.0276	up
TMEM90B	1.5445	0.0176	2.6014	0.0015	up
ZFPM2	-1.9461	0.0002	-2.3405	0.0052	down
TMEM132A	-1.7934	0.0388	-1.9598	0.0044	down
CDKN1C	-1.6331	0.0357	-2.2724	0.0083	down
NOX4	-1.3331	0.0340	-2.4069	0.0126	down
NEDD4L	-1.2668	0.0485	-1.5674	0.0431	down
PLK2	-1.2457	0.0412	-2.4713	0.0083	down
SLC25A23	-1.2426	0.0357	-1.8834	0.0149	down
SOX4	-1.0659	0.0395	-1.2718	0.0464	down

### Identification of the tissue/organ‑specific expressed genes

To identify the tissue/organ-specific expression of the DEGs in GSE39035 and GSE97311 datasets, a total of 33 tissue/organ-specific expressed genes were screened by BioGPS according to the criteria described previously (Table 3). We observed that the highest number of genes were expressed in the haematopoetic/ immune system (5/33, 15%). The second group included heart, adipocytes, and colorectal adenocarcinoma, each of which included 4 genes (4/33, 12%). The digestive system and the placenta were each represented by 3 genes (3/33, 9%) while the nervous system and genital system were represented by 2 genes (2/33, 6%). Finally, the endocrine system, respiratory system, urinary system, tongue, skeletal muscle and retina were each represented by one specifically-expressed genes (1/33 each, 3%).

**Table 3:** Distribution of tissue/organ-specific expressed genes identified by BioGPS

System/Organ	Genes	Counts
Haematologic/Immune	APBB1IP, IFIT1, TMEM140, SCIN, CDCA7	5
Placenta	CDKN1C, FBN2, TFAP2A	3
Heart	COX7A1, PPAPDC3, HSPB2, CSDC2	4
Digestive	SERPING1, CES1, CYP2E1	3
Nervous	SOX11, TMEFF2	2
Endocrine	FOXE1	1
Respiratory	ANXA3	1
urinary	NOX4	1
Genital	EMX2, HOXA11	2
Colorectal adenocarcinoma	HOXB5, KRT8, MSX1, NFE2L3	4
Others		
Adipocyte	MME, MGST1, COMP, FADS1	4
Tongue	PITX1	1
Skeletal Muscle	PDLIM3	1
Retina	PITX2	1

### Enrichment analysis

**Fig. 2:**
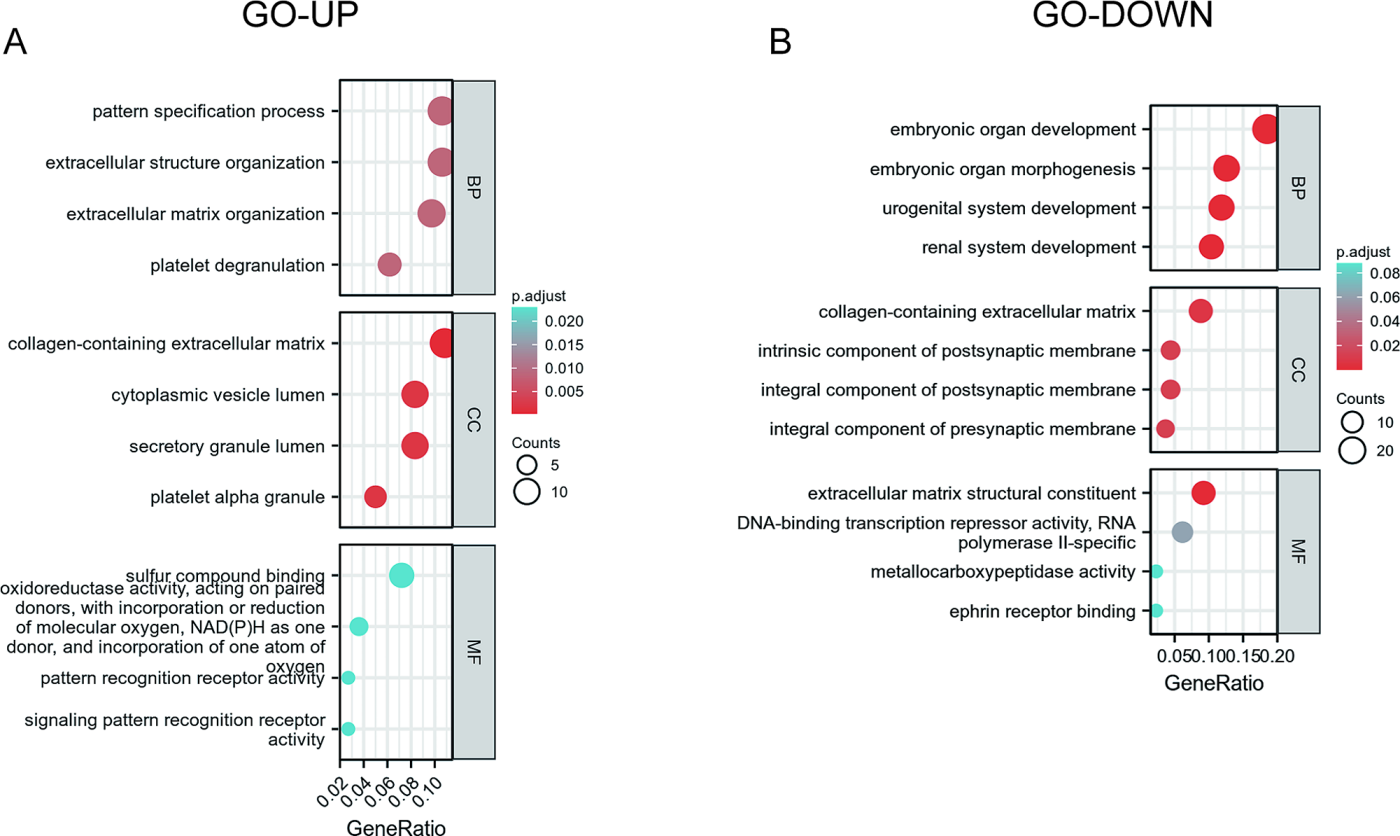
GO analysis results of co-expressed genes. Results of GO enrichment analysis of the DEGs that were upregulated (UP), or downregulated (DOWN). GO, Gene Ontology; MF, Molecular Function; BP, Biological Process; CC, Cellular Components; DEGs, differentially expressed genes

To further explore the biological function of the DEGs, the R package was used to perform GO enrichment analyses. In the biological process (BP) group, unregulated DEGs were primarily enriched in categories of extracellular structure organization, extracellular matrix organization and pattern specification process (Fig. 2A; Table 4). In contrast, downregulated DEGs were mostly enriched in embryonic organ development, embryonic organ morphogenesis and urogenital system development (Fig. 2B; Table 5). In the cellular component (CC) group, the collagen-containing extracellular matrix featured enrichment of both upregulated and downregulated genes, indicating that these genes may play different roles, respectively. Whereas the secretory granule lumen and cytoplasmic vesicle lumen showed upregulated genes and the downregulated DEGs were mostly enriched in the integral components of both the presynaptic and the postsynaptic membrane (Fig. 2A and B). In the molecular function (MF) group, upregulated genes were primarily enriched in sulfur compound binding, signaling pattern recognition receptor activity and pattern recognition receptor activity, while downregulated DEGs were mostly enriched in extracellular matrix structural constituent, DNA-binding transcription repressor activity, RNA polymerase II-specific and metallocarboxypeptidase activity (Fig. 2A and B).

**Table 4:** Significant enriched GO analysis of UP DEGs

Ontology	ID	Description	GeneRatio	pvalue	p.adjust
BP	GO:0043062	extracellular structure organization	12/113	9.59e-06	0.009
BP	GO:0002576	platelet degranulation	7/113	1.28e-05	0.009
BP	GO:0030198	extracellular matrix organization	11/113	1.45e-05	0.009
BP	GO:0007389	pattern specification process	12/113	1.66e-05	0.009
CC	GO:0062023	collagen-containing extracellular matrix	13/120	1.18e-06	2.63e-04
CC	GO:0031091	platelet alpha granule	6/120	1.96e-05	0.002
CC	GO:0034774	secretory granule lumen	10/120	2.71e-05	0.002
CC	GO:0060205	cytoplasmic vesicle lumen	10/120	4.20e-05	0.002
MF	GO:0016709	oxidoreductase activity, acting on paired donors, with incorporation or reduction of molecular oxygen, NAD(P)H as one donor, and incorporation of one atom of oxygen	4/111	1.02e-04	0.023
MF	GO:1,901,681	sulfur compound binding	8/111	1.79e-04	0.023
MF	GO:0008329	signaling pattern recognition receptor activity	3/111	2.53e-04	0.023
MF	GO:0038187	pattern recognition receptor activity	3/111	2.94e-04	0.023

**Table 5:** Significant enriched GO analysis of Down DEGs

Ontology	ID	Description	GeneRatio	pvalue	p.adjust
BP	GO:0048568	embryonic organ development	25/135	5.47e-16	1.54e-12
BP	GO:0048562	embryonic organ morphogenesis	17/135	3.12e-11	4.39e-08
BP	GO:0001655	urogenital system development	16/135	2.18e-09	2.05e-06
BP	GO:0072001	renal system development	14/135	2.81e-08	1.98e-05
CC	GO:0062023	collagen-containing extracellular matrix	12/136	2.52e-05	0.006
CC	GO:0099056	integral component of presynaptic membrane	5/136	1.50e-04	0.012
CC	GO:0099055	integral component of postsynaptic membrane	6/136	1.61e-04	0.012
CC	GO:0098936	intrinsic component of postsynaptic membrane	6/136	2.02e-04	0.012
MF	GO:0005201	extracellular matrix structural constituent	12/130	2.80e-09	8.20e-07
MF	GO:0001227	DNA-binding transcription repressor activity, RNA polymerase II-specific	8/130	4.22e-04	0.062
MF	GO:0046875	ephrin receptor binding	3/130	9.96e-04	0.088
MF	GO:0004181	metallocarboxypeptidase activity	3/130	0.001	0.088

Supplementary Table 1. Primers used for qPCR

**Fig. 3:**
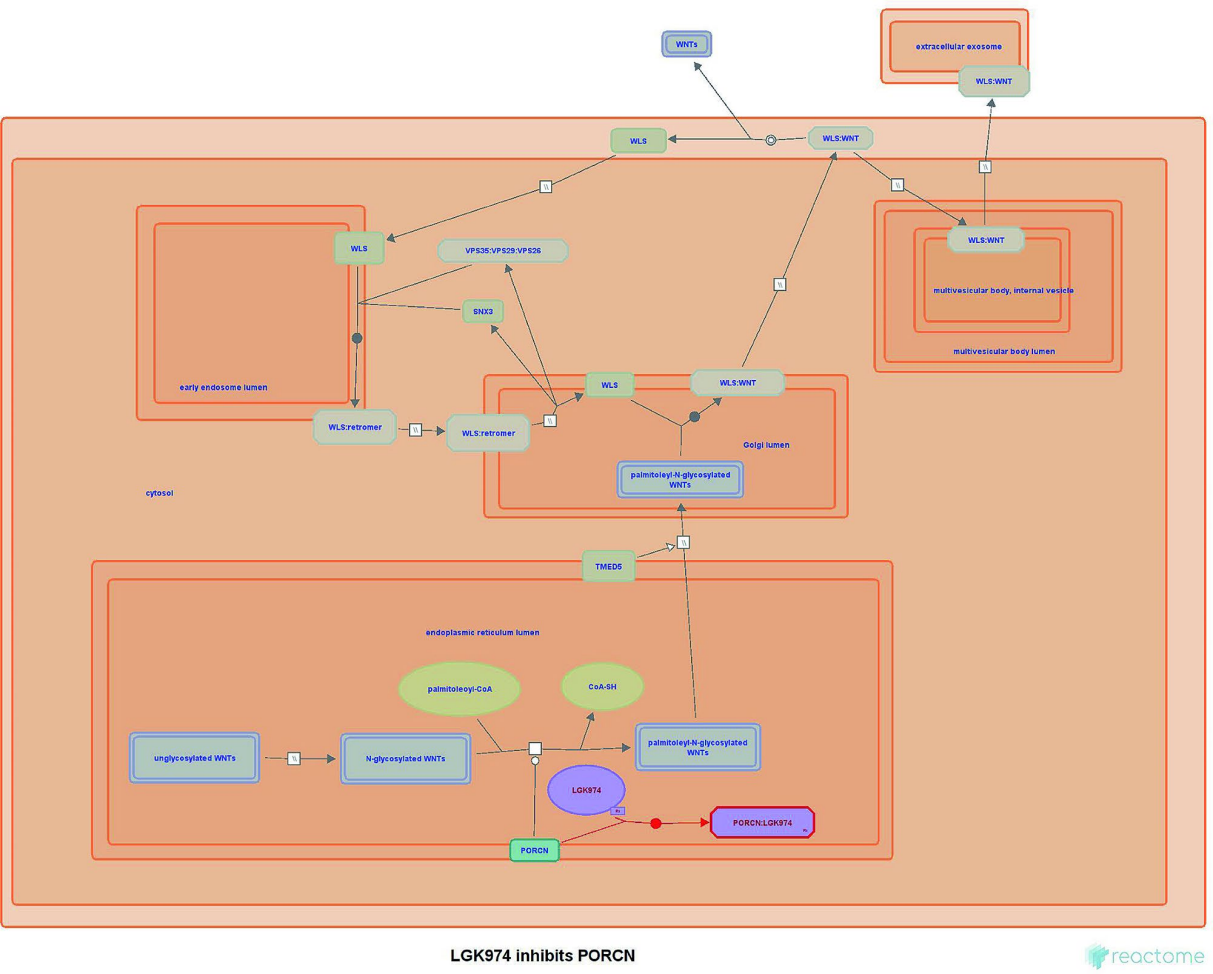
Reactome pathway enrichment analysis

In addition to GO enrichment analysis, functional enrichment analysis of DEGs were further performed using Reactome pathway database. The results showed that the upregulated DEGs were significantly enriched in the WNT signaling pathway (Fig. 3). PORCN (Porcupine O-Acyltransferase), one of the upregulated DEGs both in GSE39035 and GSE97311 datasets, is related to the WNT signaling pathway. Gene Ontology (GO) annotations related to PORCN include acyltransferase activity and palmitoleoyltransferase activity. It is well known that WNT pathway is critical for stem cell differentiation and development. Moreover, aberrant WNT signaling is associated with the development of numerous cancers [33]. LGK974, also shown on Fig. 3, is a PORCN-inhibitor that interferes with the WNT/β-catenin pathway ligands, and can be used for the treatment of WNT-dependent cancers [34].

### PPI Network analysis and hub gene identification

**Fig. 4:**
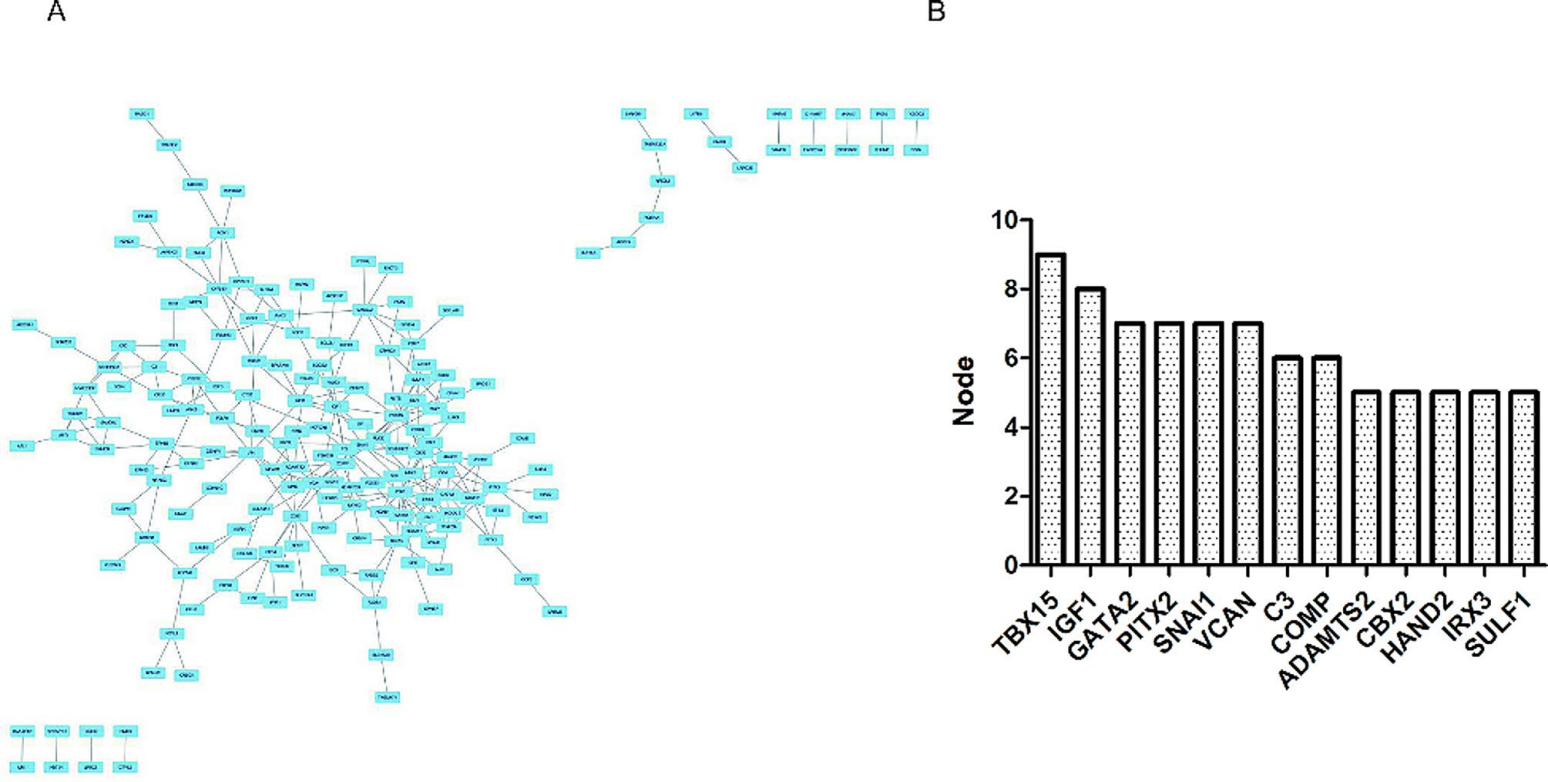
A. PPI network of DEGs in samples of MSCs from young (< 65 years of age) and old (> 65) donors. PPI, protein- protein interaction; DEGs, differentially expressed genes. B. Top 13 nodes of PPI networks of DEGs in the samples of MSCs from young (< 65 years of age) and old (> 65) donors

To explore the biological characteristics of these DEGs, a PPI network was created using the STRING database. Cytoscape was used to present the PPI network modules. As shown in Fig. 4A and B, TBX15 (T-Box Transcription Factor 15), IGF1 (Insulin Like Growth Factor 1), GATA2 (GATA Binding Protein 2), PITX2 (Paired Like Homeodomain 2), SNAI1 (Snail Family Transcriptional Repressor 1), VCAN (Versican), C3 (Complement C3), COMP (Cartilage Oligomeric Matrix Protein), ADAMTS2 (ADAM Metallopeptidase With Thrombospondin Type 1 Motif 2), CBX2 (Chromobox 2), HAND2 (Heart And Neural Crest Derivatives Expressed 2), IRX3 (Iroquois Homeobox 3), SULF1 (Sulfatase 1) proteins interact with other proteins by > 4, which was the central node of the protein interaction network. These genes are the most important genes in PPI network and may play important roles in the aging of MSCs. Finally, we chose the top six genes for further analysis.

### Validation of diagnostic value of hub genes

**Fig. 5:**
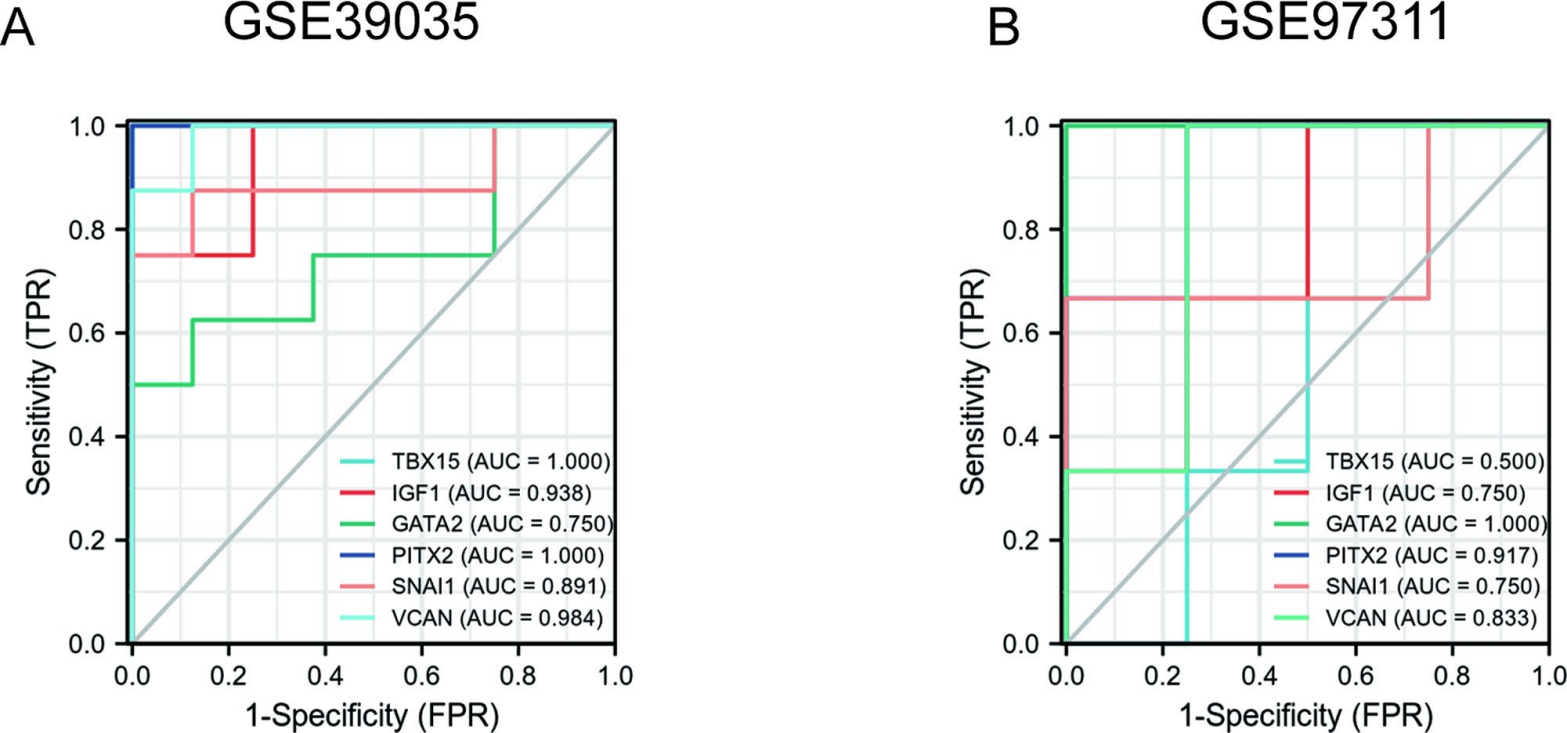
ROC curve of the hub genes in samples of MSCs from young (< 65 years of age) and old (> 65) donors. Diagnostic value of top 6 hub genes with ROC curves in GSE39035 dataset (A) and in GSE97311 dataset (B). AUC area under the ROC curve

Analysis using area under the curve (AUC), which combines both sensitivity and specificity and so can be used to describe the intrinsic effectiveness of diagnostic tests [35] was applied. To validate the diagnostic value of the top six hub genes from the above analyses, we constructed ROC curves and calculated the corresponding AUC of these gene expression levels in the GSE39035 and GSE97311 datasets. Figure 5A shows the results of GSE39035: the AUC for TBX15, IGF1, GATA2, PITX2, SNAI1, VCAN were 1.000 [95% confidence interval (Cl), 1.000–1.000; *P* < 0.05], 0.938 (95% Cl, 0.824-1.000; *P* < 0.05), 0.750 (95% Cl, 0.488-1.000; *P* < 0.05), 1.000 (95% Cl, 1.000–1.000; *P* < 0.05), 0.891 (95% Cl, 0.700-1.000; *P* < 0.05) and 0.984 (95% Cl, 0.941-1.000; *P* < 0.05). Figure 5B shows the AUC curve in the groups classified as young and old in the GSE97311 datasets. The AUC for six hub genes were 0.500 (95% Cl, 0.000–1.000; *P* < 0.05), 0.750 (95% Cl, 0.328-1.000; *P* < 0.05), 1.000 (95% Cl, 1.000–1.000; *P* < 0.05), 0.917 (95% Cl, 0.686-1.000; *P* < 0.05), 0.750 (95% Cl, 0.234-1.000; *P* < 0.05), 0.833 (95% Cl, 0.468-1.000; *P* < 0.05). These results indicate that TBX15, IGF1, GATA2, PITX2 SNAI1 and VCAN may serve as potential biomarkers for evaluating the age of MSCs donors or the quality of MSCs.

### Comparison of hub genes expressions between normal and tumor tissues

**Fig. 6:**
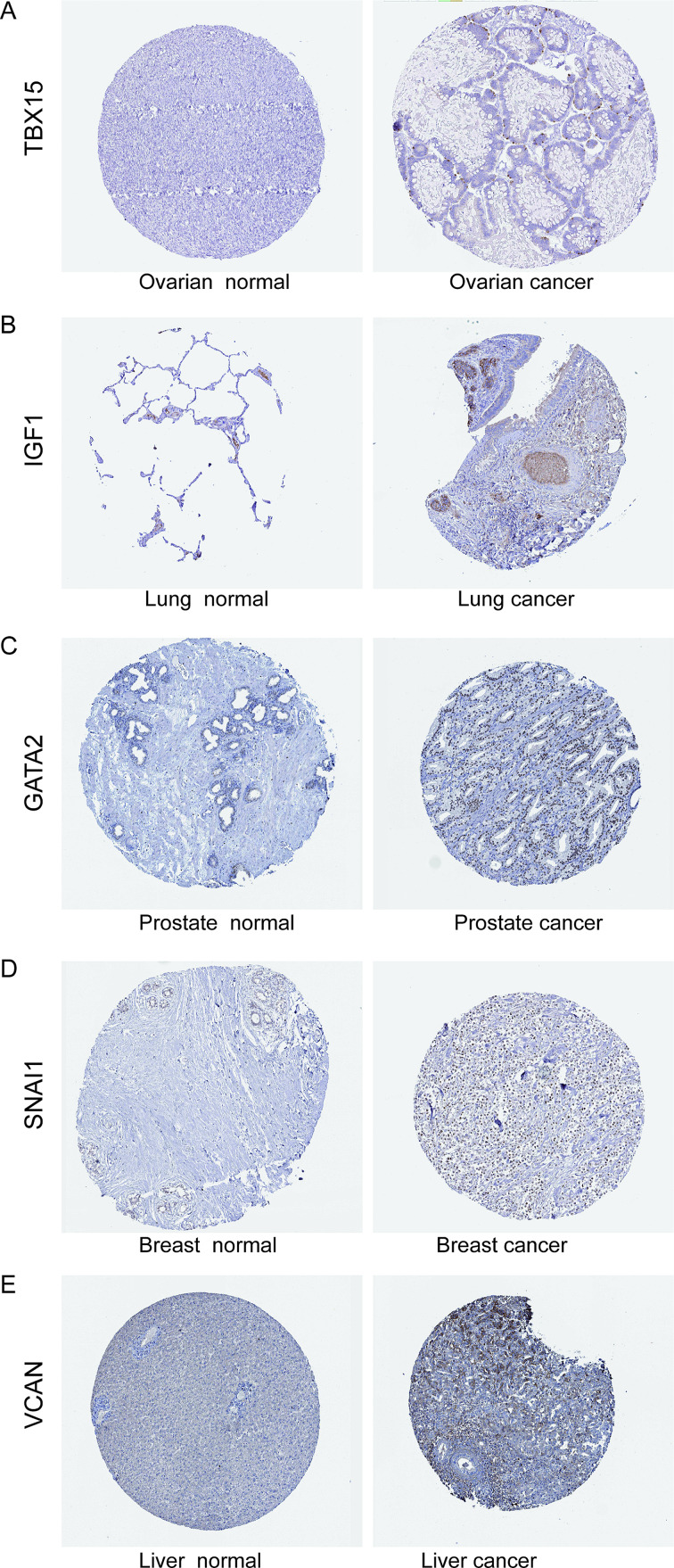
Immunohistochemistry images of hub genes in normal and tumor tissues extracted from the HPA (http://www.proteinatlas.org/). TBX15 protein expression was significantly higher in ovarian cancer tissue than normal tissue. IGF1 protein expression was significantly higher in lung cancer tissue than normal tissue. GATA2 protein expression was significantly higher in prostate cancer tissue than normal tissue. SNAI1 protein expression was significantly higher in breast cancer tissue than normal tissue. VCAN protein expression was significantly higher in liver cancer tissue than normal tissue

To evaluated hub gene (TBX15, IGF1, GATA2, SNAI1 and VCAN) expression at the protein level, the IHC images were download from the HPA database (http://www.proteinatlas.org/). As shown in Fig. 6A-E, normal ovarian, lung, prostate, breast and liver had negative or moderate IHC staining, while tumor tissues had strong staining. Furthermore, we also noted that these genes also showed high expression in other tumor tissues, such as colorectal and pancreatic (not shown). Some hub genes (TBX15 and VCAN) are not well known, but have been reported to be highly expressed in many tumors in recent years, such as gliomas [36], ovarian cancer [37]. The other hub genes, IGF1, GATA2 and SNAI1, have been reported to be widely expressed and involved in the development of many tumors [38–40]. Therefore, the results indicate that these hub genes may be involved in the development of several tumors, which is consistent with the results of our previous bioinformatics analysis.

### Validation of hub genes expression in senescent MSCs

**Fig. 7:**
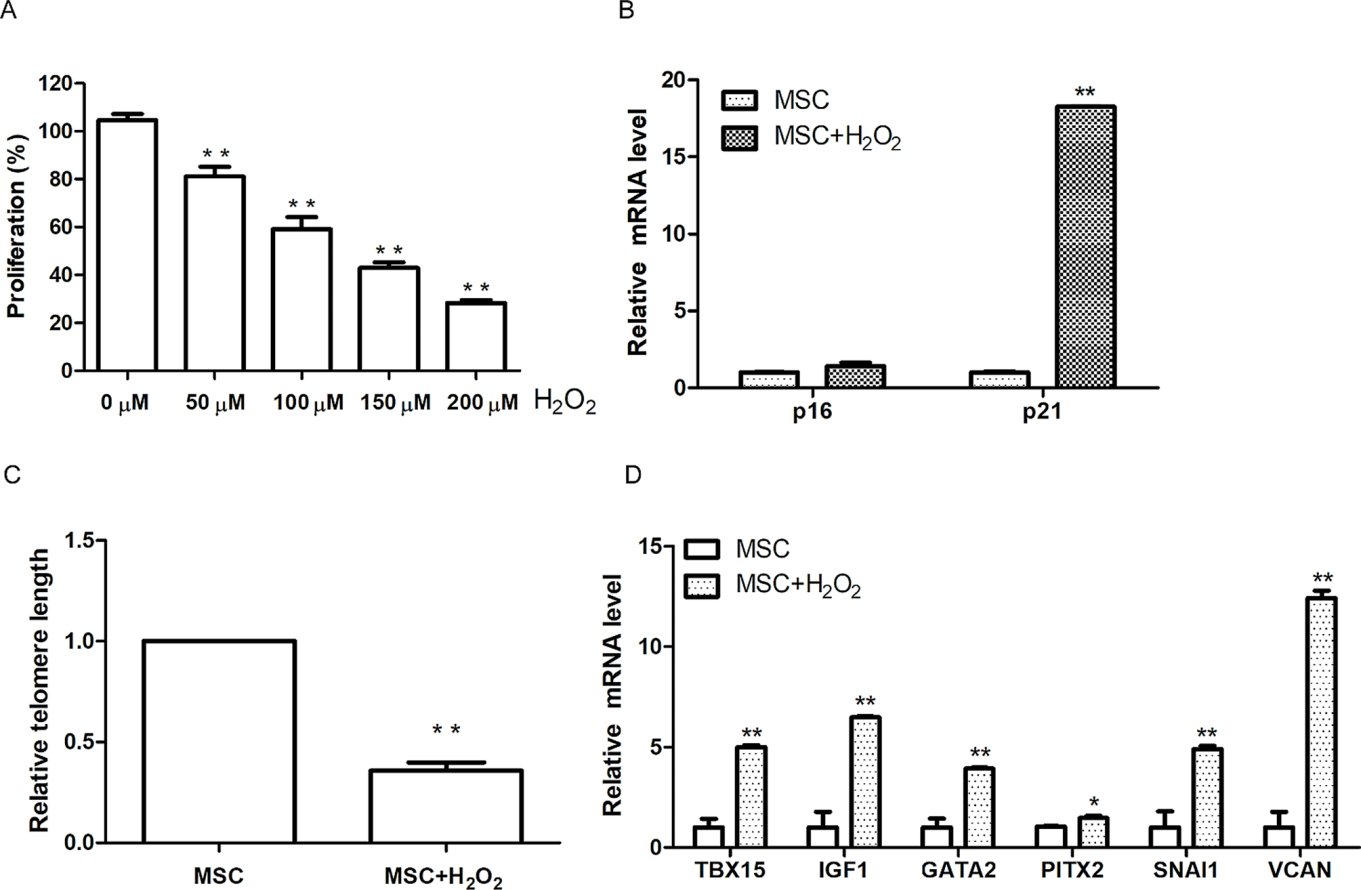
The expression of hub genes in H_2_O_2_ treated MSCs

To further assess whether hub genes can be used as diagnostic markers for senescence in MSCs donors, we cultured human bone marrow-derived MSCs in vitro and induced cellular senescence with different concentrations of H_2_O_2_. As shown in Fig. 7A, the viability of MSCs was significantly reduced in a concentration-dependent manner after H_2_O_2_ treatment. We chose 100 µM H_2_O_2_ to treat MSCs for 2 h and extracted total mRNA to detect changes in the expressions of senescent markers p16 and p21. As shown in Fig. 7B, we observed that expressions of both senescence markers, p16 and p21, was significantly upregulated in MSCs after H_2_O_2_ treatment. Moreover, by comparing the relative telomere lengths in MSCs and MSC-H_2_O_2_ groups, we further confirmed that the relative telomere length was significantly shortened in MSCs after H_2_O_2_ treatment (Fig. 7C). These results indicated that MSCs entered senescence state following induction by H_2_O_2_. Subsequently, we used qPCR analysis to detect the expression of the six hub genes in the H_2_O_2_-induced MSCs at the transcriptional level (Fig. 7D**)**. Compared with the control MSCs group, the expression of all six hub genes, including TBX15 (*p* < 0.01), IGF1 (*p* < 0.01), GATA2 (*p* < 0.01), PITX2 (*p* < 0.05), SNAI1 (*p* < 0.01) and VCAN (*p* < 0.01) (*p* < 0.01), was significantly upregulated in senescent MSCs. These results suggest that the expressions of these hub genes are elevated in senescent MSCs, which is consistent with the foregoing results in this paper. It indicates that the expression of some potentially tumor-associated genes may be upregulated in senescent MSCs donors compared to young donors, and thus there may be a higher safety risk of using MSCs from old donors to treat diseases.

## Discussion

In the emerging field of cell therapy, MSCs therapy has attracted attention because of the unique properties, stemness, and immunomodulatory ability of the MSCs. MSCs are considered as a ‘safer source’ for cell therapy with minimal risk that transplanted MSCs will form tumors and become cancerous, while possessing a low level of immunogenicity [41]. Therefore, MSC therapy is considered to have considerable clinical potential. However, both pre-clinical and clinical studies have provide mixed results for MSC therapy, and the discrepancy between expected and actual efficacy of MSCs in various diseases has evoked a sense of discouragement [42]. One likely reason behind this is the source of MSCs, especially the age of the donor, and the culture conditions.

The aging of MSCs leads to an age-associated decline in their number and functions including declines in multilineage differentiation, homing, immune modulation, and wound healing [41]. Some researchers have suggested that ageing of MSCs is recognized as an important culprit in organismal aging [43]. Some studies have also reported that MSC senescence is closely related to the occurrence of various diseases, including malignant tumors. For example, this is especially true in adult MSCs, which accumulate cancer-driving mutations with age [44]. Age-related MSC dysfunction is particularly evident in barrier epithelia of aging organisms, resulting in dysplasias, degenerative diseases, and cancers [45]. However, studies about aging and MSC therapy have focused mainly on in vitro MSC aging itself, or so called ‘replicative senescence’ [41]. In contrast, little is known about the impact of the origin of MSCs, especially the effect of donor age on the function and gene expression of MSCs.

In our study, we identified 337 DEGs, including 33 tissue/organ-specific expressed genes, by comparing genes expressed in old and young samples from the GSE39035 and GSE37311 databases. We found that DEGs, such as CDKN1C, NEDD4L, PLK2 and SOX4, were closely related to the occurrence and development of malignant tumors. For example, CDKN1C as a prognostic biomarker correlated with immune infiltrates and therapeutic responses in breast cancer patients [46]. NEDD4L inhibits oral cancer proliferation by inducing ENO1 ubiquitination and degradation [47]. PLK2 served as a novel biomarker for the prognosis of human glioblastoma [48]. SOX4 regulates invasion of bladder cancer cells via repression of WNT5a [49].

In addition, the differential expression of ZFPM2 (Zinc Finger Protein, FOG Family Member 2), PORCN (Porcupine O-Acyltransferase), NOX4 (NADPH Oxidase 4) and SLC25A23 (Solute Carrier Family 25 Member 23) plays important roles in various diseases [50–52].

Meanwhile, some reports have suggested that PORCN and SOX4 are closely related to the WNT signaling pathway [50–53]. In this respect, it has been suggested that the Wnt/β-catenin signaling pathway is also involved in the regulation of MSC senescence [54], which is consistent with our results.

Moreover, comparing the samples from young and old donors, four DEGs (HOXB5, KRT8, MSX1, NFE2L3) in the samples from old donors were specifically expressed in colorectal adenocarcinoma (4/33, 12%) (Table 3) as noted above. Many reports have suggested that these four genes are differentially expressed in colorectal adenocarcinoma [55–57]. These are consistent with our findings, suggesting that MSCs derived from aged donors may carry a higher risk of carcinogenesis. This may explain to some extent why MSCs therapy may cause various diseases and even malignant tumors.

In this study, we identified the 13 top genes by constructing a PPI network and screening hub genes. The top six hub genes TBX15, IGF1, GATA2, PITX2, SNAI1, VCAN were validated by ROC curves in both the GSE39035 and GSE97311 datasets.

GATA2, a key transcription factor, is required to preserve hematopoietic stem cell function [58]. It has been reported that GATA2 is the target of ZFPM2, which could be activated or down-regulated. Our finding also confirmed that ZFPM2 was significantly down-regulated in samples from older compared with younger donors in the two datasets. Consistent with this research, we found that ROC curve of GATA2 seemed to have a very high diagnostic value in samples from the older group of donors (AUC = 0.750; AUC = 1.000) (Fig. 5). Hence, we consider GATA2 has potential as an effective biomarker for diagnosis of age-compromised MSCs.

Diseases associated with SNAI1 include some malignant tumors. It has been suggested that SNAI1 participate in stem cell self-renewal [59]. Consistent with our study, we found that the AUC of ROC curve of SNAI1 were 0.89 (GSE39035) and 0.75 (GSE97311), respectively, indicating that SNAI1 might have potential as a biomarker for diagnosis of the age status of donor MSCs. Similarly, TBX15, IGF1, PITX2 and VCAN appear to exhibit a high diagnostic potential in both the GSE39035 and GSE97311 datasets.

There may also be another opportunity to de-risk the use of MSCs derived from older donors by pre-treatment of such sub-optimal MSCs with exosomes from the MSCs of more potent donors, such as from young people, or from an animal-derived source. In this latter case, deer antlers may provide one such source. For example, Lei et al. (2021) reported that treatment with antler stem cell-derived exosomes effectively attenuated senescent phenotypes in human MSCs, probably by promoting self-renewal and repressing the senescence-related genes (such as P16 and P21, and IL-8, IL-1β etc.) [60]. In addition to the age, donor genetic backgrounds, lifestyle and diet also influence the function of MSCs [17, 61]. In the future, these factors should also be taken into account before treating diseases using MSCs.

## Conclusions

Through the use of public databases, bioinformatics analysis and experimental validation, we have confirmed that MSCs derived from aged donors exhibit differential expression of some genes compared with those from young donors; such changes may affect the function of MSCs, and may induce malignant tumors. We identified two co-expressed UP genes and eight co-expressed DOWN genes from the databases of both GSE39035 and GSE97311. We further confirmed that six genes (TBX15, IGF1, GATA2, PITX2, SNAI1 and VCAN) may have potential as biomarkers for the diagnoses of the aging state of donor MSCs, and thus provide a basis and reference for the clinical application of MSCs to treat diseases. Taken together, these results have prompted us to develop clearer criteria for MSCs donor sources for clinical MSCs therapy.

MSCs were treated with different concentrations (0, 50, 100, 150 and 200 µM) of H_2_O_2_ for 2 h, and then cultured 48 h. Cell viability was measured using CCK-8 kit (A). Total RNA and DNA from MSCs and MSCs treated with H_2_O_2_ (100 µM) were isolated respectively. Relative mRNA expressions of p16 and p21 were quantified by qRT-PCR (B). Relative telomere lengths were determined by qPCR (C). Relative mRNA expressions of hub genes (TBX15, IGF1, GATA2, PITX2, SNAI1 and VCAN) were quantified by qRT-PCR (D). Data are normalized to the value of MSCs group. Data are presented as means ± SD. *P < 0.05; **P < 0.01.

### Electronic supplementary material

Below is the link to the electronic supplementary material.

Supplementary Material 1

## Data Availability

The datasets analyzed in this study are available in GEO database. GSE39035 repository at https://www.ncbi.nlm.nih.gov/geo/query/acc.cgi?acc=GSE39035. GSE97311 repository at https://www.ncbi.nlm.nih.gov/geo/query/acc.cgi?acc=GSE97311.

## Declarations

### Author details

^1^ Scientific Research Center, China-Japan Union Hospital of Jilin University, Changchun 130000, Jilin, China.

^2^ Life Spring AKY Pharmaceuticals, Changchun 130000, Jilin, China.

^3^ Institute of Antler Science and Product Technology, Changchun Sci-Tech University, Changchun 130000, Jilin, China.
