# Supplementary figures and images for: Identification of potential biomarkers for aging diagnosis of mesenchymal stem cells derived from the aged donors
Source: Stem Cell Res Ther. 2024 Mar 22;15:87. doi: 10.1186/s13287-024-03689-1 (PMC10960456; doi:10.1186/s13287-024-03689-1)

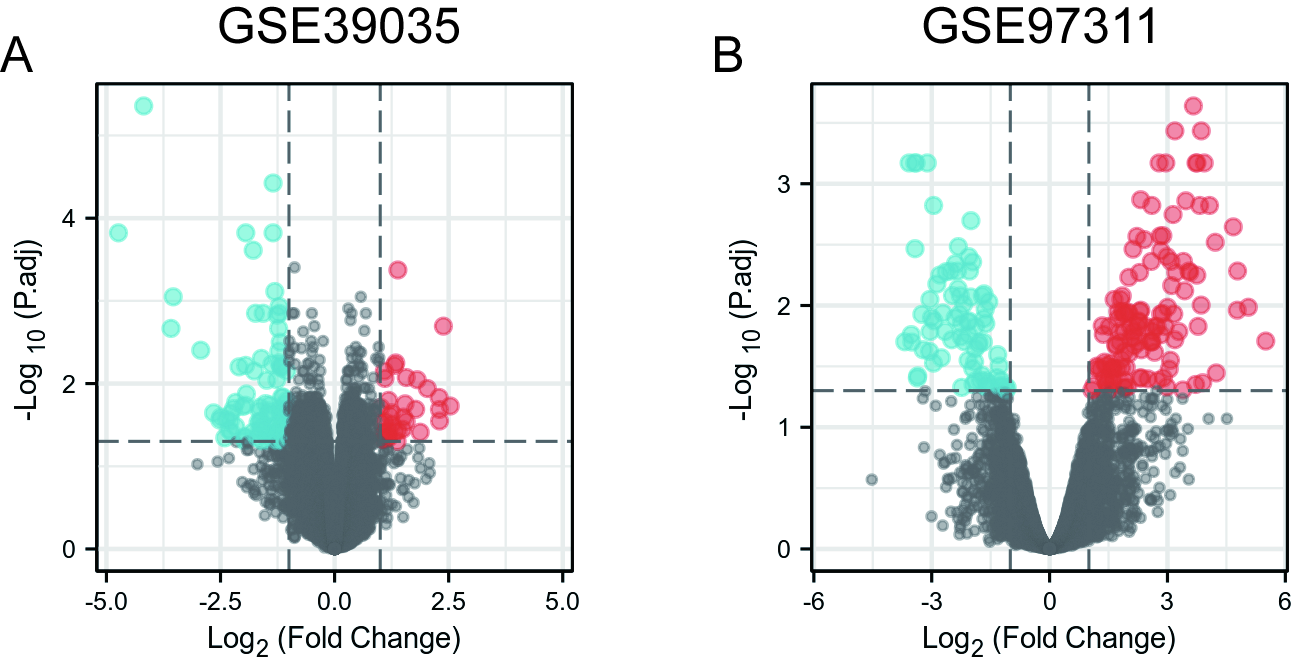

Supplement: Supplementary file 1 — Supplementary Material 1 [file 13287_2024_3689_MOESM1_ESM.tif]
